# Effects of non-pharmaceutical interventions on COVID-19 transmission: rapid review of evidence from Italy, the United States, the United Kingdom, and China

**DOI:** 10.3389/fpubh.2024.1426992

**Published:** 2024-10-17

**Authors:** Laura J. Faherty, Pedro Nascimento de Lima, Jing Zhi Lim, Derek Roberts, Sarah Karr, Emily Lawson, Henry H. Willis

**Affiliations:** ^1^RAND Corporation, Boston, MA, United States; ^2^Maine Medical Center, Portland, ME, United States; ^3^Tufts University School of Medicine, Boston, MA, United States; ^4^RAND Corporation, Arlington, VA, United States; ^5^RAND Corporation, Santa Monica, CA, United States; ^6^RAND Corporation, Pittsburgh, PA, United States

**Keywords:** COVID-19, non-pharmaceutical interventions, effective reproduction number, contact rate, disease transmission, infectious disease modeling

## Abstract

**Background:**

Prior to the development of COVID-19 vaccines, policymakers instituted various non-pharmaceutical interventions (NPIs) to limit transmission. Prior studies have attempted to examine the extent to which these NPIs achieved their goals of containment, suppression, or mitigation of disease transmission. Existing evidence syntheses have found that numerous factors limit comparability across studies, and the evidence on NPI effectiveness during COVID-19 pandemic remains sparse and inconsistent. This study documents the magnitude and variation in NPI effectiveness in reducing COVID-19 transmission (i.e., reduction in effective reproduction rate [R_eff_] and daily contact rate) in Italy, the United States, the United Kingdom, and China.

**Methods:**

Our rapid review and narrative synthesis of existing research identified 126 studies meeting our screening criteria. We selected four contexts with >5 articles to facilitate a meaningful synthesis. This step yielded an analytic sample of 61 articles that used data from China, Italy, the United Kingdom, and the United States.

**Results:**

We found wide variation and substantial uncertainty around the effectiveness of NPIs at reducing disease transmission. Studies of a single intervention or NPIs that are the least stringent had estimated Reff reductions in the 10–50% range; those that examined so-called “lockdowns” were associated with greater Reff reductions that ranged from 40 to 90%, with many in the 70–80% range. While many studies reported on multiple NPIs, only six of the 61 studies explicitly used the framing of “stringency” or “mild versus strict” or “tiers” of NPIs, concepts that are highly relevant for decisionmakers.

**Conclusion:**

Existing evidence suggests that NPIs reduce COVID-19 transmission by 40 to 90 percent. This paper documents the extent of the variation in NPI effectiveness estimates and highlights challenges presented by a lack of standardization in modeling approaches. Further research on NPI effectiveness at different stringency levels is needed to inform policy responses to future pandemics.

## 1 Introduction

In the early months of the COVID-19 pandemic, while clinical trials of candidate vaccines were underway, policymakers instituted a wide range of non-pharmaceutical interventions (NPIs), which are defined as strategies intended to limit transmission until a vaccine and other pharmaceutical countermeasures are available to the public (1). These NPIs included various forms of social/physical distancing; such as shelter-in-place orders (often called “lockdowns”), school closures, bans on mass gatherings, mandatory indoor masking, testing requirements, contact tracing, and quarantine (1, 2).

These measures, while implemented for the protection of public health, were costly and disruptive to society (3). Evidence on the extent to which these NPIs “worked,” (i.e., achieved their public health goals of containment, suppression, or mitigation of disease transmission) (4), was urgently needed. Although policymakers and researchers often prefer to rely on evidence from randomized trials, challenges to estimating the effects of NPIs on clinical outcomes abound. These include endogeneity (i.e., policymakers introduce NPIs dynamically in response to COVID-19 case data), lack of consistency (i.e., individual NPIs can be combined in numerous ways and implemented with different levels of stringency and adherence), and the impossibility of randomly assigning individuals to most interventions other than masks. As a result, estimating the impact of NPIs on clinical outcomes is often infeasible and, at best, is done after the estimate is no longer decision-relevant. Given the challenges of conducting randomized studies to answer this critical question, researchers typically used observational data (e.g., historical or geographical) and quasi-experimental methods such as interrupted time-series analysis, and infectious disease modeling (1) to estimate the impact of NPIs on outcomes such as COVID-19 transmission, cases, or deaths. Notable exceptions are two pragmatic randomized studies of the effectiveness of masking. One cluster-randomized study, conducted in Bangladesh, found that “mask wearing averaged 13.3% in villages where no interventions took place but increased to 42.3% in villages” where free masks were distributed in conjunction with promotion efforts, and in villages randomized to mask-wearing interventions, symptomatic seroprevalence was reduced by about 35%; the other study, from Denmark, randomized at the individual level, and results were inconclusive regarding whether an individual recommendation to wear a mask was associated with infection (5, 6).

Following an explosion of studies examining NPI effectiveness, there have been recent efforts to synthesize existing studies of the impact of NPIs on COVID-19 outcomes (1), including a series of rigorous evidence reviews focused on specific NPI types (e.g., masks and face coverings, social distancing and “lockdowns;” test, trace, and isolate) (7–12). These evidence reviews identified substantial variation in COVID-19 modeling approaches and concluded that this variation limits comparability across different studies. An accompanying series of case studies on Hong Kong, New Zealand, and South Korea demonstrated that it was possible to control transmission with early, stringent NPI implementation but that as naturally- or vaccine-induced immunity increased, all three countries relaxed their interventions and moved from suppression to mitigation of transmission (13). While these case studies yield important lessons for policymakers, they cannot be directly generalized to other contexts. Thus, public health practitioners and policymakers at all levels, from national to local, continue to make decisions based on data from contexts that do not reflect their specific situation or produce widely varying estimates of NPI effectiveness.

Furthermore, studies that use absolute observed outcomes such as avoidance of cases, hospitalizations, or deaths as their primary outcomes are common. In fact, they represent one-third of studies from the first year of the pandemic (14) despite these outcomes being biased by factors such as testing availability, inpatient capacity and healthcare infrastructure, and variation in case reporting and categorization of deaths associated with COVID-19. Infectious disease modelers continue to have limited and inconsistent evidence to draw on when parameterizing their models. To avoid model misinterpretation, experts have called for assessing effectiveness of NPIs using epidemiological parameters that measure relative changes in person-to-person transmission at a population level (4, 14).

Focusing on two key limitations of existing research on NPI effectiveness: imprecise definitions of health outcomes and limited attention to local contextual factors when drawing conclusions about these measures, this study builds on prior evidence syntheses. Specifically, we document the variation in NPI effects on COVID-19 transmission measures in four selected geographies: China, Italy, the United States, and the United Kingdom. Despite the challenges of synthesizing existing evidence, we offer suggested next steps for modelers and policymakers.

## 2 Methods

### 2.1 Search strategy

To guide our search strategy, we focused on the two main epidemiological measures of disease transmission: time-varying effective reproduction number (often denoted as R_e_ or R_eff_) and contact rate, typically denoted as b or β (beta).

The study team formulated database search strategies for PubMed (National Institute of Health/National Library of Medicine) and Web of Science Core Collection (Clarivate). Within the Web of Science Core Collection, the search was limited to the following databases: *Science Citation Index Expanded* (SCI-EXPANDED), *Social Sciences Citation Index* (SSCI), and *Emerging Sources Citation Index* (ESCI). This was to decrease the number of unwanted publication types, such as conference proceedings and book chapters, and results from unrelated research areas, such as Arts & Humanities. The following limits were applied to each database search: Articles in peer-reviewed literature, available in English language, and published between November 2019–June 2023.

The primary search was conducted in PubMed and was then translated for Web of Science. The research question was divided into 6 main concepts (search strings) that were combined using Boolean operators: COVID-19, Non-Pharmaceutical Interventions (NPIs), Transmission Prevention and Precautions, Infectious Contacts, Basic Reproduction Number, and Study Type. The Basic Reproduction Number search string was created to increase the yield of relevant studies that estimated the effect of NPIs on quantities versus studies that were estimating the interventions on the number of COVID-19 cases and/or deaths. Study Type was included to narrow the scope of results to the desired type of publications using the PubMed-provided “Article Type” Filters, relevant Medical Subject Headings (MeSH) terms, and the field tag for Title/Abstract ([tiab]).

The searches were conducted on August 2, 2023. The search results from both databases were saved as RIS files and then imported into an EndNote library for de-duplication and to assign details to the records, including database source and the search date. Appendix A contains details on all search strategies.

### 2.2 Article screening procedures

**Figure 1:**
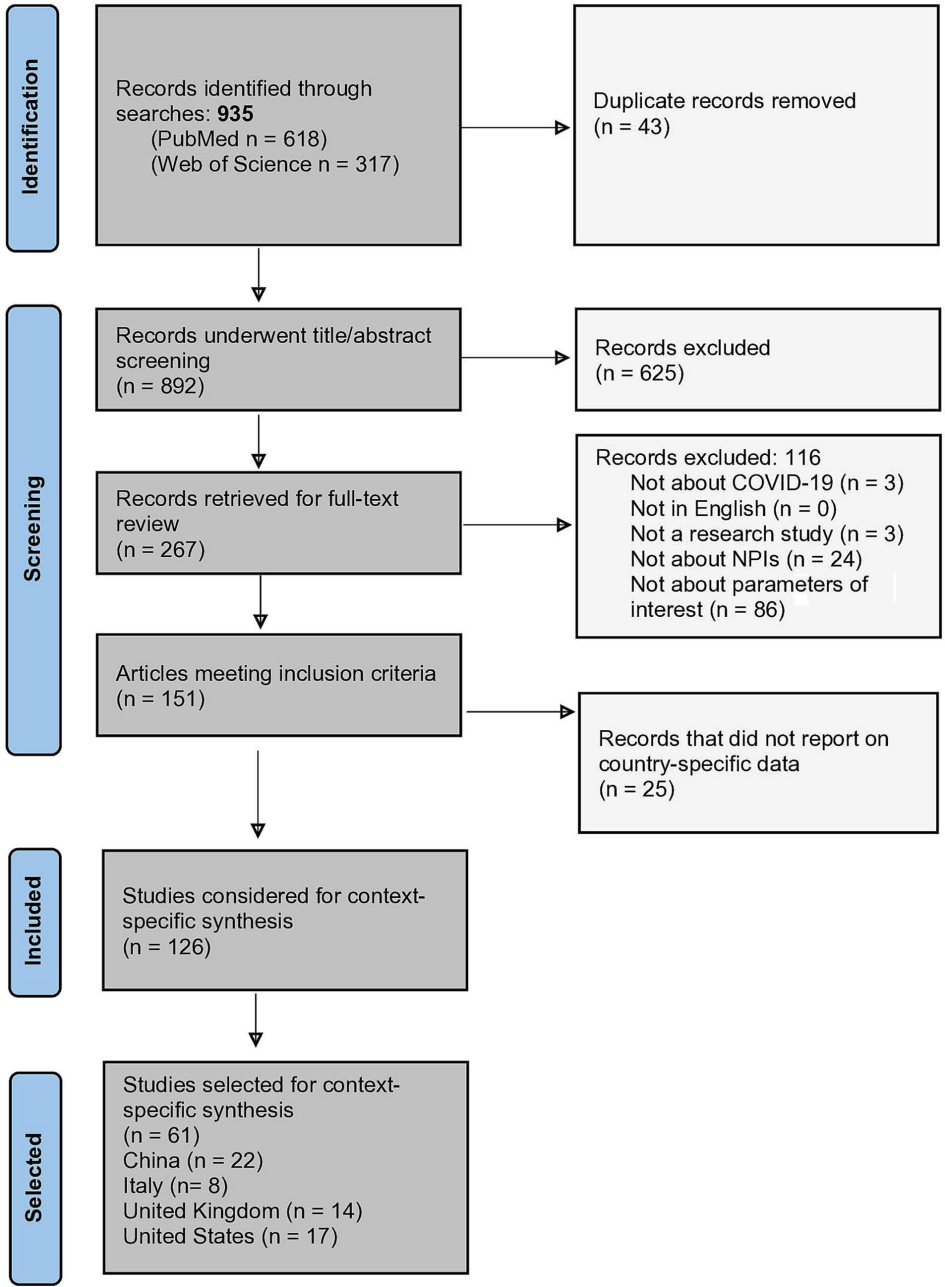
PRISMA flowchart (24).

In the first stage of screening, we reviewed article titles and abstracts for inclusion. The study team excluded articles that met the following criteria: (1) not about COVID-19; (2) not in English; (3) not a report of primary research (e.g., commentaries, letters to the editor); (4) not about NPIs (e.g., only about vaccination); (5) outcomes were not our epidemiological parameters of interest (e.g., only included estimates of cases, deaths). After the initial title and abstract screening, articles were reviewed in full by one reviewer, then approximately 50% underwent a second review by LJF to yield a sample of 151 articles (Figure 1).

### 2.3 Data abstraction

Using a structured Excel form, the study team abstracted standardized information from each article, following an adapted version of the PICOTS framework (16). As articles rarely reported on the populations of interest, the comparators are assumed to be the counterfactual scenario in which NPIs were not implemented, and the outcomes of interest were pre-specified as an inclusion criterion, the remaining relevant elements of PICOTS were (1) intervention(s; NPIs); (2) time frame of the study; and (3) setting in which the research was conducted.

### 2.4 Selection of contexts

Studies were categorized by country or countries of focus. Hereafter, we use the term “contexts” to indicate either a single country or a group of politically affiliated countries (e.g., the United Kingdom) that are often reported on as a unit. An additional 25 articles that did not present context-specific data were excluded at this step given the study’s emphasis on synthesizing evidence within a specific context, which yielded a sample of 126 included articles (Figure 1). Finally, the study team selected four contexts with enough articles to facilitate a meaningful synthesis, defined as >5 articles: China, Italy, the United Kingdom, and the United States.

### 2.5 Synthesis of findings

To synthesize this literature, we completed a descriptive summary of the articles that used data from each selected country or context. We attempted to synthesize findings by looking for patterns within and across each country/context. However, this was not possible due to the diversity of methodologic approaches and modeling assumptions and lack of consistency about the populations examined, the interventions themselves, and other dimensions of PICOTS.

### 2.6 Compiling summary information on COVID-19 disease burden and policy responses

Once our four contexts were selected, we conducted a targeted literature review and web-based scan for country-specific COVID-19 policies in Italy, the United States, the United Kingdom, and China, as well as data on COVID-19 cases and deaths by the end of 2020 (17). Using publicly available data from the University of Oxford’s COVID-19 Government Response Tracker (18), we calculated the median score on the Health and Containment Index (HCI) for each of the four countries for the year 2020. We synthesized this information to provide context for the findings from the literature search.

## 3 Results

Of the 127 studies included in the review, our final analytic sample consisted of 61 articles, 22 using data from China; 8 using data from Italy; 17 using data from the United States, and 14 using data from the United Kingdom.

### 3.1 Italy

**Figure 2:**
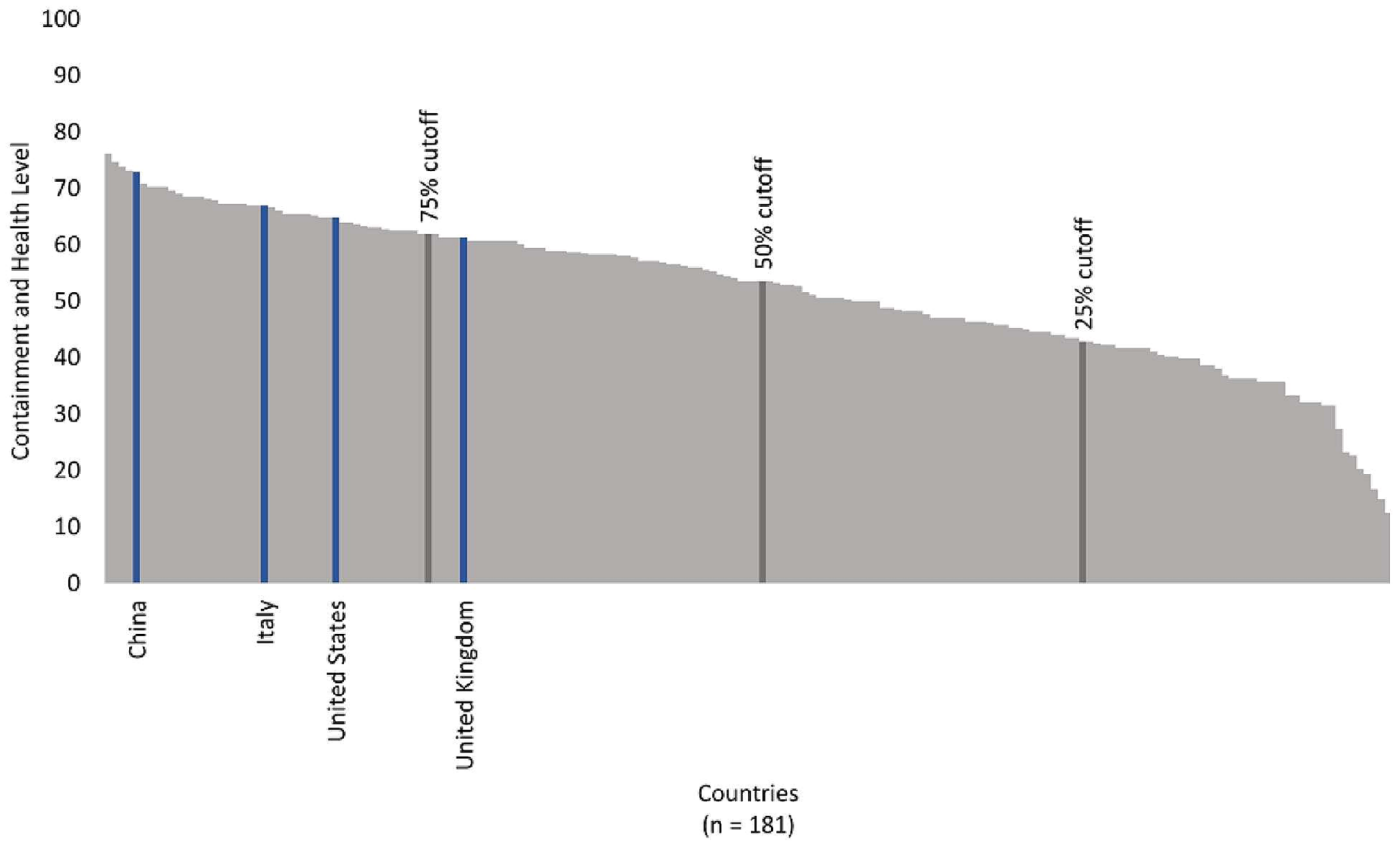
Median country containment and health levels, 2020, for countries with available data, indicating the four selected contexts for this analysis in relation to the 75th percentile cutoff.

Italy was the first European country to report a case of COVID-19, which occurred on January 31, 2020 (19). It was also one of the most severely impacted, with 2,141,201 confirmed cases and 74,985 deaths by the end of 2020 (20), corresponding to about 1,247 deaths per million (17). Shortly after Italy’s first COVID-19 case was detected, policymakers instituted a national stay-at-home policy during the months of March and April 2020 (21, 22). This policy was in place during the first wave (February–May 2020). Then, in response to an increase in incidence that signaled the beginning of Italy’s second wave, which lasted from October to December 2020, the government instituted a three-color classification system on November 4 that guided regional approaches to NPIs. Italy’s median HCI score for 2020 was 67, slightly higher than the United States and well above the 75th percentile cutoff for all countries (Figure 2).

We identified eight studies using data from Italy that met our inclusion criteria, six of which exclusively reported data from Italy and two of which were studies of multiple countries that included case data from Italy (Table 1). Two used primary data collected through surveys. The studies reflected interventions, time frame, and setting in the following manner:

- **Intervention(s)**: Four studies examined the effects of social distancing or “lockdowns” on R_eff_ (23–26); and four studies examined multiple NPIs in combination (27–30).
- **Time frame**: Nearly all studies used February or March 2020 as the start of the study time frame, and five examined data from only a few months (e.g., February to March 2020; or February to May 2020). Two of the eight studies’ data extended into 2021 (28, 30).
- **Setting**: Studies were primarily conducted at the national level in Italy, with the exceptions of one that focused on Milan (30) and one on Lombardy (24).

**Table 1:** Summary of studies of NPI effects on transmission parameters using data from Italy, from largest to smallest percentage reduction in effective reproduction number (*N* = 8).

Article information	Context		NPI(s) examined	Study type	Key findings			
Author, Year	Time frame	Location if specified			% reduction in R_eff_	Minimum R_eff_(t)	Pre-intervention R_0_	% reduction in β
Albi, 2021 (23)	Mar-May 2020	–	Social distancing/“lockdown”	Model-based	~96^a^	~0.2	~4.9	–
Del Fava, 2021 (27)	Mar-Apr 2020	–	Multiple	Survey-based	85	~0.4	~2.65	88.1
Marziano, 2021 (26)	Feb-Sep 2020	–	Social distancing/“lockdown”	Model-based	~80	~0.7	~2.7	~80
Mohammadi, 2022 (28)	Feb 2020-Jan 2021	–	Multiple	Model-based	74	~0.5	4.05	~44
Guzzetta, 2021 (25)	Mar-Apr 2020	–	Social distancing/“lockdown”	Model-based	~74	0.76	2.83–3.10	–
Armaou, 2022 (24)	Feb-May 2020	Lombardy	Social distancing/“lockdown”	Model-based	~70	~1.1	4.38	–
Gatto, 2020 (29)	Feb-Mar 2020	–	Multiple	Model-based	45	–	3.60	–
Trentini, 2022 (30)	Jul 2020-Mar 2021	Milan	Multiple	Survey-based	17, 25, 45^b^	–	–	16, 30, 42

a Tildes (“~”) denote approximate values that were not presented in the text of an article and therefore were estimated from a figure.

b Percent reductions in the case of least stringent, more stringent, and most stringent NPIs, respectively.

Among the eight studies that reported changes in R_eff_, the estimated percentage reduction in this parameter typically ranged from about 45% (29) to nearly 96 percent (23), with several studies reporting a percent reduction in the 70–80% range. One of these eight studies reported three different percentage reductions in R_eff_ by level of NPI stringency, estimating a 44.7% reduction only with the most stringent set of interventions. When interventions were implemented with medium stringency, the authors estimated a 25.1% reduction in R_eff_ and that percentage was only 17.1% with the least stringent set of NPIs (30) (Table 1).

Four of the eight studies reported percentage reductions in daily contact rates, ranging from about 16 to 88%. One of those studies, (the same analysis that reported different reductions in R_eff_ for three levels of NPI stringency), also reported three different reductions in daily contact rates which were again lower than other studies from Italy, finding that they decreased by 16 with the least stringent suite of NPIs, 30% with an intermediate stringency level, and 42% with the most stringent set (30).

### 3.2 United States

The first COVID-19 case in the United States was confirmed on January 20, 2020, (31) and by the end of 2020, there were 20,271,441 cases and 362,570 deaths (20), corresponding to about 1,057 deaths per million (17). Like Italy, the United States aimed for suppression of viral transmission (32). States implemented stay-at-home orders, mask mandates, inter-state travel restrictions, and other policies to contain the spread of COVID-19 during March and April 2020. After states began to reopen in May and June 2020, the country experienced a second wave of cases, then saw a third wave in October 2020. The United States’ median HCI score in 2020 was 65 (Figure 2).We identified 17 studies that met our inclusion criteria and used data from the United States, only two of which collected primary data from surveys or passive monitoring (Table 2).

**Table 2:** Summary of studies of NPI effects on transmission parameters using data from the United States, from largest to smallest percentage reduction in effective reproduction number.

Article information	Context		NPI(s) examined	Study type	Key findings			
Author, Year	Time frame	Location if specified			% reduction in R_eff_	Minimum R_eff_(t)	Pre-intervention R_0_	% reduction in β
Ngonghala, 2020 (42)	Feb–Dec 2020	New York State	Multiple	Model-based	90	0.20	1.95	–
Pei, 2020 (77)	Mar–May 2020	–	Multiple	Model-based	89	0.34	3.10	–
Yang, 2021 (78)	Jan–Jul 2020	–	Multiple	Model-based	86	0.65	4.80	–
Kim, 2022 (41)	Jun 2020–May 2021	New York City	Multiple	Model-based, survey	83	0.50	3.08	–
Buchwald, 2020 (33)	Jan–Nov 2020	Colorado	Social Distancing	Model-based	83	0.9	5.3	76%
Childs, 2021 (37)	Apr–Jun 2020	Santa Clara County, California	Quarantine	Model-based	83	0.64	3.76	–
Yang, 2021 (79)	Mar–Jul 2020	New York City	Multiple	Model-based	81	0.56	2.99	–
Aleta, 2020 (34)	Feb–Dec 2020	Boston	Social Distancing	Model-based	75	~ < 1	~ > 4	–
Lau, 2022 (43)	Mar–June 2020	Georgia	Multiple	Model-based	59	–	–	–
Olney, 2021 (80)	Feb–Apr 2020	–	Multiple	Model-based	52	0.88	1.86	–
Kain, 2021 (35)	Feb–Oct 2020	See footnote^a^	Social Distancing	Model-based	50–75	<1	~2–4	–
Goyal, 2021 (39)	Mar–Aug 2020	King County, Washington	Masking	Model-based	33	1	1.5	–
Brook, 2021 (40)	Aug 2020–Apr 2021	University setting^b^	Testing	Model-based	22–83	–	–	–
Unwin, 2020 (38)	Feb–Jun 2020	–	Quarantine	Survey, passive monitoring	15	–	–	–
Dreher, 2021 (81)	Jan–Apr 2020	–	Multiple	Observational study	13	1.09	1.26	5–15%
Guo, 2021 (36)	Jan–Jul 2020	–	Social Distancing	Observational study	12	0.88	1.2	–
Xie, 2022 (82)	Mar–Nov 2020	–	Multiple	Model-based	See note^c^		–	

a Los Angeles and Santa Clara Counties, California; Seattle (King County), Washington; Atlanta (Dekalb, Fulton Counties), Georgia; and Miami (Miami-Dade County), Florida.

b Using model inputs derived from the authors’ experience with the University of California—Berkeley student population.

c This study presented the reduction in reproduction numbers as coefficients with standard errors. As no baseline R_0_ was reported, it was not possible to calculate the percentage reduction in R_eff_.

These studies varied by intervention(s) examined, time frame of the study, and setting(s) in which they were conducted, as follows:

- **Intervention(s)**: Nine studies looked at multiple NPIs simultaneously, four studies examined the impact of social distancing on R_eff_ (33–36), two studies focused on quarantine (37, 38), one study focused on the effects of masking (39), and one study explored the impact of testing (40).
- **Time frame**: Most of the studies used data collected during the first wave of the COVID-19 pandemic in the United States, with 15 of the study time frames beginning in the first 4 months of 2020, most ending in the late summer/early fall 2020, and only two extending into 2021 (40, 41).
- **Setting**: Three used data from a single state (33, 42, 43), five used data from a single county or city (34, 37, 39, 41), and one study was conducted in a university setting (40). The others used national-level data, included data from multiple cities or counties (35), or did not specify a setting.

These studies using data from the United States found widely varying estimates of our two key outcomes of interest, R_eff_ and daily contact rate. The estimated percentage reduction in R_eff_ ranged from 12% (36) in a study evaluating social distancing to approximately 90% (42) in an analysis of multiple NPIs in combination (Table 2). Overall, studies that found a percent decrease in R_eff_ that was less than 50% tended to be studies of a single NPI such as masking or testing, while those that found greater reductions tended to be studies of multiple NPIs in combination. Otherwise, the variation in assumptions, modeling approaches, and data sources precludes further comparisons of the key findings.

### 3.3 United Kingdom

The United Kingdom’s first COVID-19 case was reported on January 30, 2020 (44), and by the end of 2020, there were 2,722,251 cases and 95,706 deaths in this country, corresponding to approximately 1,079 deaths per million (17). While the United Kingdom initially took a mitigation approach (i.e., aiming to minimize the pandemic’s impacts on vulnerable groups to avoid overwhelming the health care system while permitting controlled transmission in low-risk groups) (32), it shifted to a suppression strategy in April 2020 as daily incidence rates increased (32). Public health measures were relaxed in June 2020 but were reinstituted in response to a second wave in September 2020. The United Kingdom’s median HCI score in 2020 was 61, putting it just below the 75^th^ percentile cut point for all countries with available data (Figure 2).

We identified a total of 14 studies using data from the United Kingdom that met our inclusion criteria (Table 3). These studies varied across interventions, time frame, and setting as follows:

- **Intervention(s)**: Eight studies examined multiple containment measures in combination, three studies examined “lockdowns” (45, 46), one evaluated the impact of social distancing (47); one evaluated testing, tracing, quarantine, and isolation (48); and one study examined work-from-home policies (49).
- **Time frame**: The majority of studies (*n* = 12) were conducted at the peak of the first wave of COVID-19 in 2020. Three were performed during the nascent stages of the pandemic from February to April 2020, preceding the UK government’s shift to a more stringent suppression strategy in April 2020 (47, 50, 51). Only one study extended into 2021 (46), and another extended to February (52).
- **Setting**: Ten studies looked at the United Kingdom broadly, while four limited their analysis to data from England only (46, 52, 53).

**Table 3:** Summary of studies of NPI effects on transmission parameters using data from the United Kingdom, from largest to smallest percentage reduction in effective reproduction number.

Article information	Context		NPI(s) examined	Study type	Key findings			
Author, Year	Time frame	Location if specified			% reduction in R_eff_	Minimum R_eff_(t)	Pre-intervention R_0_	% reduction in β
Hill, 2021 (49)	2020	–	Work from home	Model-based	93	~0.2	~3	–
Morgan, 2021 (83)	Feb–Jul 2020	–	Multiple	Model-based	~92	~0.2	2.8	60%
Challen, 2021 (45)	Feb–Jul 2020	–	Lockdown	Model-based	~77	0.70	~ 3.1	–
Knock, 2021 (53)	Mar–Dec 2020	England	Multiple	Model-based, survey, passive monitoring	76	0.68	2.8	–
Jarvis, 2020 (47)	March 2020	–	Social distancing	Survey	76	0.62	2.60	74%
Brooks-Pollock, 2021 (84)	Jan–Jun 2020	–	Multiple	Survey	74^a^	0.7	2.7	–
Perez-Guzman, 2023 (52)	Mar 2020–Feb 2022	England	Multiple	Model-based	71	0.75	2.6	–
Kucharski, 2020 (50)	Feb–Apr 2020	–	Multiple	Model-based	64	0.91	2.6	–
Davies, 2020 (85)	Feb–Oct 2020	–	Multiple	Model-based	62	< 1	2.7	–
Van Bunnik, 2021 (51)	Feb 2020	–	Multiple	Model-based	50	~0.8	~1.6	75%
Davies, 2021 (86)	Mar–Oct 2020	England	Lockdown	Model-based	44	–	–	–
Eales, 2022 (46)	Jan–Jul 2021	England	Lockdown	Model-based	30	0.76	1.08	–
Grassly, 2020 (48)	Mar–Jun 2020	–	Test and trace	Model-based	26	–	–	–
Laydon, 2021 (54)	Jul–Nov 2020	–	Multiple	Model-based	6 (Tier 2), 23 (Tier 3)	–	1.3	–

^aHere we report the maximal reduction, observed with lockdown procedures.^

The reduction in R_eff_ varied substantially across the studies, with estimates ranging from as low as 6% (54) considering tier 2 restrictions to a reduction of approximately 93%, from a simulation study of work from home policies (49) (Table 3). Similar to the studies from the United States, studies from the United Kingdom that reported smaller decreases in R_eff_ typically examined the impact of less stringent measures, either a single NPI or the lowest-tier set of interventions, while those reporting greater R_eff_ reductions generally evaluated more stringent interventions such as “lockdowns” or the combined effect of multiple NPIs.

### 3.4 China

After Chinese authorities published the sequence of the novel coronavirus, SARS-CoV-2 (55) on January 12, 2020, Chinese health officials instituted one of the strongest policy responses of any country with the goal of completely interrupting transmission, commonly termed a “Zero-COVID” strategy (56). This aggressive containment strategy (32) involved strongly-enforced stay-at-home orders, including through the extensive use of electronic health passes; multiple rounds of mass screening testing for entire cities; contact-tracing involving global positioning system (GPS) technology; and a host of other NPIs (e.g., mask mandates, screening inbound travelers) that were rapidly implemented and sustained throughout the first year of the pandemic (57). By the end of 2020, China had reported 96,972 cases and 4,791 deaths (20), corresponding to 3.2 deaths per million people (17). China’s median HCI score over the course of 2020 was 73, one of the highest of any country with available data, only exceeded by Argentina, Chile, Peru and Oman (Figure 2).

We identified a total of 22 studies using data from China that met our inclusion criteria (Table 4). These studies presented interventions, time frame, and setting in the following manner:

- **Intervention(s)**: Six studies examined social distancing or lockdown measures, one focused on school closure, and the remaining 15 examined the effects of suites of non-pharmaceutical interventions.
- **Time frame**: Most (*N* = 16) included studies were centered in 2020. Several used data starting December 2019 (Wuhan-based). Only six study time frames extended beyond 2020 and only one used 2023 data (58).
- **Setting**: The majority of studies were conducted at the city level. Five analyzed the initial Wuhan outbreak, two focused on Beijing, and others used data from Shanghai (59), and Guangzhou (60). The remainder compared cities across China to one another or used data from Hong Kong.

**Table 4:** Summary of studies of NPI effects on transmission parameters using data from China, from largest to smallest percentage reduction in effective reproduction number (*N* = 22).

Article information	Context		NPI(s) examined	Study type	Key findings			
Author, Year	Time frame	Location if specified			% reduction in R_eff_	Minimum R_eff_(t)	Pre-intervention R_0_	% reduction in β
Studies reporting effective reproduction number and/or contact rate								
He, 2022 (65)	Dec 2020–Feb 2021	–	Social distancing/“lockdown”	Model-based	~99, ~96	~0.05, ~0.1	3.63, 2.45^a^	–
Xia, 2020 (87)	Jan–Apr 2020	–	Multiple	Model-based	98	0.1	~4.6	–
Cai, 2022 (60)	Apr 2022	Guangzhou	Multiple	Model-based	98	0.1	4.20	–
Liu, 2021 (66)	Jan–Mar 2020	Wuhan	Multiple	Model-based	97	0.1	3.86	–
Yang, 2021 (88)	Dec 2019–Feb 2020	–	Multiple	Model-based	95	0.24	5.16	–
Zhou, 2021 (89)	May 2021–Jan 2022	–	Multiple	Model-based	~94, ~95, ~90	~0.3, ~0.2, ~0.4	4.93, 3.73, 3.71^b^	–
Hao, 2020 (67)	Jan–Mar 2020	Wuhan	Social distancing/“lockdown”	Model-based	92	0.28	3.54	–
Leung, 2020 (90)	Jan–Feb 2020	–	Multiple	Model-based	~92, ~64	~0.1, ~0.25	~1.3, ~0.7^c^	–
Yang, 2020 (91)	Feb–Mar 2020	–	Multiple	Model-based	91, 82	0.46, 0.92^d^	5.16	–
Chen, 2022 (68)	Jan–Feb 2020	Wuhan	Social distancing/“lockdown”	Model-based	87	0.33	2.5	–
Zeng, 2022 (61)	May–Jun 2021	–	Multiple	Model-based	~87	~0.5	~4.0	77.4^e^
Yang, 2020 (69)	Jan–Feb 2020	Wuhan	Multiple	Model-based	84	1.13	6.98	–
Leung, 2023 (58)	Nov 2022–Jan 2023	Beijing	Multiple	Passive monitoring	79	0.72	3.44	–
Cowling, 2020 (92)	Feb–Mar 2020	Hong Kong	School closure	Survey-based	77	~0.5	~2.2	–
Zhang, 2021 (62)	Mar–May 2020	–	Multiple	Survey-based	77, 5^f^	–	–	–
Lei, 2021 (70)	Jan–Mar 2020	–	Multiple	Passive monitoring	66, 48, 35^g^	–	2.12	–
Cui, 2021 (93)	Jun–Jul 2020	Beijing	Social distancing/“lockdown”	Model-based	55	3.0	6.6	–
Li, 2022 (94)	Dec 2019–Apr 2020	–	Multiple	Passive monitoring	52, 63	0.12, 0.06	0.25, 0.17^h^	–
Fang, 2020 (64)	Dec 2019–Apr 2020	–	Multiple	Model-based	36, 5, 39^i^	–	–	–
Hu, 2023 (59)	Mar–Apr 2022	Shanghai	Multiple	Model-based	24	1.3	1.7	–
Studies only reporting contact rate								
Huang, 2021 (63)	Dec 2019–May 2020	Wuhan	Social distancing/“lockdown”	Model-based	–	–	–	~98^j^
Zhang, 2020 (95)	Jan–Feb 2020	–	Social distancing/“lockdown”	Survey-based	–	–	–	86.9, 74.5^k^

a Haerbin and Shijiazhuang, respectively.

b Xi’an, Yangzhou, and Guangzhou, respectively.

c Shanghai and Beijing, respectively.

d With strict interventions, with “mild interventions” respectively.

e Reduction due to phase II social distancing interventions.

f Reduction from school closure and halting of community activities and reduction from school closure only, respectively.

g Total reduction, estimated reduction in response to quarantine, and estimated reduction in response to social distancing, respectively.

h Primary cases and secondary cases, respectively.

i Reductions due to intracity transport restrictions alone, intercity transport restrictions alone, and non-movement restrictions, respectively.

j “Total social contacts index reduction” in Wuhan.

k Wuhan and Shanghai, respectively.

These studies reported a wide range of NPI effectiveness. Only three studies examined changes in daily contact rate; percent reduction ranged from 75 to 98 (61–63) (Table 4). All studies aside from three reported percent reduction in the effective reproductive number (R_eff_) or pre-intervention R_eff_ and minimum R_eff_ from which percent reduction could be extrapolated. Percent reductions ranged from 5%, in studies that only examined inter-city transport restrictions (64) or school closure (62), to 99% (65), in a study of lockdown. However, most studies reported large reductions in R_eff_, e.g., 11 studies reported percent reductions of 90% or more. Among all cites examined, NPI effectiveness was highest in Wuhan: 84–97% reductions in R_eff_ (63, 66–69) and a 98% reduction in contact rate (63).

## 4 Discussion

We conducted a rapid review and narrative synthesis of context-specific evidence to document the magnitude and variation of NPI effectiveness during the pre-vaccine phase of the COVID-19 pandemic. Our analysis of recent published studies from four countries found wide variation and substantial uncertainty around the NPI effectiveness estimates, despite narrowing the focus to two epidemiological parameters rather than outcomes such as cases, hospitalizations, and deaths, and despite examining studies from the same local context. In addition, we found that while many studies of the 61 we examined reported on multiple different NPIs, either individually or in combination, only six studies explicitly used the framing of “stringency” or “mild versus strict” or “tiers” of NPIs, which are concepts that are highly relevant for decisionmakers (30, 54, 62, 64, 70).

Existing literature suggests that NPIs are highly effective at reducing disease transmission. However, studies report a wide range of estimated effectiveness, defined in our study as reductions in the time-varying reproduction number, due primarily to variation in NPI definitions, time frames, and settings. Overall, studies of a single intervention or NPIs that are the least stringent estimated R_eff_ reductions in the 10–50% range, while those that examined “lockdowns,” shorthand for the most stringent social distancing measures or stay-at-home orders, were associated with greater R_eff_ reductions that ranged from 40 to 90%, with many clustered around the 70–80% range.

The challenges we encountered align with those reported by Lison et al., who concluded that a lack of standardized practices for intervention assessment, outcome reporting, and modeling frameworks has limited the utility of existing evidence (4). For example, many of the studies that met our inclusion criteria defined social distancing as broad “lock-downs” while others focused more narrowly on a specific type of physical/social distancing measure, such as working from home. Some examined an NPI in (relative) isolation, like masking or testing-and-tracing, while others took a broader view of suites of NPIs that were implemented concurrently. Studies also varied in the population of interest, including students at a university or school, workers who have the ability to work from home, or the general population. Finally, studies assessed interventions across varying time frames. Data from shorter study periods might capture immediate effects of NPIs but miss long-term trends including sustainability of adherence to those NPIs and effects of their de-implementation. On the other hand, longer time frames allow for data on these trends but results might be difficult to interpret given changing contextual factors over time.

Addressing this lack of standardization would enable the global community to share insights on the relative effectiveness of different, but well-defined NPIs (alone and in combination) across countries where effects are expected to be similar, a key recommendation from a previous report (4). Until better, more standardized data are available, modelers seeking to incorporate NPI effectiveness into their pandemic projections should strive to consider scenarios that reflect different combinations of NPIs, speed, and stringency of their implementation, expected public adherence, and timing of de-implementation. They should also ensure that they are clearly communicating the broad uncertainty these factors create for their modeling parameters.

The results of NPI effectiveness we summarize are not causal estimates, and the wide range of effectiveness estimates within and across countries highlights the degree of uncertainty that is an inherent challenge to this complex work. Yet these results are still useful to inform the debate about the benefits, drawbacks, and cost-effectiveness of NPIs for pandemic response. These relatively large effectiveness estimates reflect an unprecedented collective public health effort to reduce disease transmission during a pandemic and have a clear mechanistic rationale (i.e., less contact with other humans reduces person-to-person transmission of a respiratory pathogen). Hence, these estimates suggest societies *can* mount a collective effort to reduce disease transmission by *at least* 40%, and a 70–80% reduction in disease transmission is possible. Whether achieving those reductions is worth the adverse consequences to society including quantifiable and non-quantifiable costs, is a separate but critical question. Existing literature shows the answer is nuanced and depends on, pathogen transmissibility and severity, the anticipated time frame for developing vaccines, and the cost of NPIs relative to disease severity (71, 72). Hence, this study contributes to the literature by documenting the plausible range of one of the inputs for cost-effectiveness analyses of various NPI strategies.

Details around NPI implementation, including how quickly the measures are put into place and their degree of stringency, are key pieces of information that would be highly relevant to modelers and to decision-makers considering these NPI measures. In addition, how well the public adheres to the NPIs once they are implemented varies enormously depending on the local context, and societal trust in institutions is a strong predictor of adherence to public health recommendations (73–75). Yet, these details are mostly missing from the existing evidence base and contribute to both the mathematical uncertainty around the effectiveness estimates as well as policymaker uncertainty about how to interpret these data. Two notable exceptions are a study of mostly middle-to-high-income European countries that found that wealth and demographic structure explain country-level variation in “NPI effects” (defined as relative change in avoided new infections) (76) and the one study in our sample of 61 that estimated different reductions in the specific COVID-19 transmission parameters of interest by NPI stringency. Additional studies examining varying degrees of NPI stringency in countries with different demographic compositions and income levels are urgently needed to inform the debate about the effectiveness of NPIs.

Our findings may be subject to the following limitations: we may have missed relevant studies through our search strategy, inadvertently excluded studies that met our inclusion criteria, or misclassified information abstracted from included studies.

In summary, our work demonstrates the extent of the variation in assessments of NPI effectiveness in the early months of the COVID-19 pandemic and highlights the challenges presented by a lack of standardization in modeling approaches. To improve our global scientific preparedness, (to be ready with pre-positioned research infrastructure and protocols in advance of the next public health emergency), we must collectively address this lack of standardization, as well as other limitations articulated by Lison et al. (4). These include insufficient explanations of modeling decisions, unrealistic assumptions that influence model parameterization, and imprecise definitions of interventions and outcomes. These goals will not be simple to achieve, but they are critically important. Otherwise, when we face the next, inevitable pandemic, or a more transmissible or deadly SARS-CoV-2 variant that evades our existing vaccines, infectious disease modelers and researchers will once again produce siloed results that are not comparable within or across local contexts, and decisionmakers will be again faced with making complex policy choices based on limited evidence.

## Data Availability

The original contributions presented in the study are included in the article/Supplementary material and further inquiries can be directed to the corresponding author.
