# Supplementary material for: Effects of non-pharmaceutical interventions on COVID-19 transmission: rapid review of evidence from Italy, the United States, the United Kingdom, and China
Source: Front Public Health. 2024 Oct 17;12:1426992. doi: 10.3389/fpubh.2024.1426992 (PMC11524874; doi:10.3389/fpubh.2024.1426992)
Supplement: Supplementary file 1 [file Table_1.DOCX]

# SEARCH STRATEGIES

**PubMed**

| **Set #** | **Search** | **# of results** |
| --- | --- | --- |
| **1**  **COVID-19** | COVID*[ti] OR coronavirus*[ti] OR "corona virus*"[ti] OR "SARS CoV 2"[ti] OR SARSCoV2[ti] OR SARSCoV-2[ti] OR "2019 nCoV"[ti] OR 2019nCoV[ti] OR "nCoV 19"[ti] OR "nCoV 2019"[ti] OR "Severe Acute Respiratory Syndrome Coronavirus 2"[ti] OR COVID-19[MAJR] OR "Coronavirus"[Mesh:NoExp] OR "Coronavirus Infections"[MAJR] OR "SARS-CoV-2"[MAJR] | 357,276 |
| **2**  **NPIs** | ("non-pharmacological intervention*"[tiab] OR "nonpharmacological intervention*"[tiab] OR "nonpharmacologic intervention*"[tiab] OR "non-pharmacologic intervention*"[tiab] OR "nonpharmaceutical intervention*"[tiab] OR "non-pharmaceutical intervention*"[tiab] OR NPI[tiab] OR NPIs[tiab] OR "social distancing*"[ti] OR "social separation*"[ti] OR "social restrict*"[ti] OR "physical distancing*"[ti] OR "physical separation*"[ti] OR "spatial distancing*"[ti] OR "spatial separation*"[ti] OR "spacial separation*"[ti] OR "contact reduction*"[ti] OR "contact avoidance"[ti] OR ((house*[ti] OR home*[ti] OR self*[ti]) AND (confin*[ti] OR quarantin*[ti] OR isolat*[ti])) OR "travel restriction*"[ti] OR "travel ban"[ti] OR "travel bans"[ti] OR "banned travel"[tiab:~1] OR "mobility restriction*"[ti] OR "lockdown*"[ti] OR "lock down*"[ti] OR "shelter in place"[ti] OR "stay home"[tiab:~1] OR "remain home"[tiab:~1] OR ((school*[ti] OR college*[ti] OR university*[ti] OR business*[ti] OR workplace*[ti] OR office*[ti] OR worksite*[ti] OR "work site*"[ti]) AND (closure*[ti] OR closing*[ti] OR closed[ti] OR cancel*[ti] OR suspend*[ti] OR suspension*[ti])) OR "remote work*"[ti] OR "work home"[tiab:~2] OR ((gathering*[ti] OR crowd*[ti] OR "public event*"[ti] OR "public activit*"[ti] OR "group event*"[ti] OR "group activit*"[ti] OR "social event*"[ti] OR "social activit*"[ti] OR "nonessential activit*"[ti] OR "non essential activit*"[ti] OR "social mixing"[ti] OR gastronomy[ti] OR restaurant*[ti]) AND (restrict*[ti] OR limit*[ti] OR cancel*[ti] OR reduc*[ti] OR postpon*[ti] OR ban[ti] OR bans[ti] OR banned[ti] OR banning[ti] OR suspend*[ti] OR suspension*[ti])) OR "border clos*"[ti] OR "community containment"[tiab:~1] OR "contagion mitigat*"[ti] OR "containment measure*"[ti] OR "suppression measure*"[ti] OR "mitigation measure*"[ti] OR ((stringen*[tiab] OR strict*[tiab] OR enforcement*[tiab] OR enforcing[tiab] OR mandatory[tiab]) AND (mandate*[tiab] OR policy[tiab] OR policies[tiab] OR order*[tiab] OR law[tiab] OR laws[tiab] OR directive*[tiab])) OR "Social Isolation"[MAJR] OR "Physical Distancing"[MAJR]) | 87,577 |
| **3**  **Transmission Prevention + Precautions** | (((transmission*[ti] AND (droplet*[ti] OR aerosol*[ti] OR airborne[ti] OR "air contaminat*"[ti])) AND (prevent*[ti] OR precaution*[ti] OR protect*[ti] OR reduc*[ti] OR decreas*[ti]))) OR "cough etiquette"[ti] OR mask*[ti] OR "N95 respirator*"[ti] OR "N95 mask*"[ti] OR "hand wash*"[ti] OR "handwash*"[ti] OR "hand hygiene"[ti] OR "hand disinfect*"[ti] OR "hand sanitiz*"[ti] OR "hand sanitis*"[ti] OR "N95 Respirators"[MAJR] OR Masks[MAJR] OR "air filtration"[ti] OR "air filter*"[ti] OR "HEPA filter*"[ti] OR "air purif*"[ti] OR "air ventilat*"[ti] OR "air recirculat*"[ti] OR "Air Filters"[MAJR] OR "Ventilation"[MAJR] | 34,134 |
| **4**  **Contacts** | transmission*[tiab] OR transmissib*[tiab] OR "community spread*"[tiab] OR superspread*[tiab] OR super-spread*[tiab] OR "infectious contact*"[tiab] OR "contact pattern*"[tiab] OR "contact mapping"[tiab] OR "mixing pattern*"[tiab] OR "incubation period"[tiab] OR "Disease Transmission, Infectious"[MAJR] OR "Infectious Disease Incubation Period"[MAJR] OR "Contact Tracing"[MAJR] | 519,496 |
| **5**  **Basic Reproduction Number** | “reproduction number"[tiab] OR "reproductive number"[tiab] OR R_0[tiab] OR R0[tiab] OR R-0[tiab] OR R_0_[tiab] OR R*_0_*[tiab] OR Beta_t[tiab] OR "effective contact rate*"[tiab] OR "contact trac*"[tiab] OR "transmission tracing*"[tiab] OR "trace strateg*"[tiab] OR "contact survey*"[tiab] OR "contact matrices"[tiab] OR "contact matrix"[tiab] OR "contact mapping"[tiab] OR "reduction estimate"[tiab:~1] OR CoMix[tiab] OR Polymod[tiab] OR "susceptible exposed infectious recovered"[tiab:~2] OR SEIR*[tiab] OR "susceptible exposed asymptomatic infectious recovered"[tiab] OR SEAIR*[tiab] OR "compartmental model*"[tiab] OR ((model*[tiab] OR Theoretical Models[Mesh:NoExp]) AND (outbreak*[tiab] OR epidemic*[tiab] OR pandemic*[tiab])) OR "predictive model*"[tiab] OR forecast*[tiab] OR "Basic Reproduction Number"[MAJR] OR "Epidemiological Models"[MAJR] OR "Basic Reproduction Number"[Mesh] OR "Epidemiological Models"[Mesh] | 416,954 |
| **6** | #1 AND (#2 OR #3) AND #4 AND #5 | 1,356 |
| **7**  **Study Type** | (comparativestudy[Filter] OR evaluationstudy[Filter] OR governmentpublication[Filter] OR meta-analysis[Filter] OR observationalstudy[Filter] OR researchsupportamericanrecoveryandreinvestmentact[Filter] OR researchsupportnonusgovt[Filter] OR researchsupportusgovtnonphs[Filter] OR researchsupportusgovtphs[Filter] OR researchsupportusgovernment[Filter] OR review[Filter] OR systematicreview[Filter] OR technicalreport[Filter] OR "epidemiologic report*"[tiab] OR "epidemiologic stud*"[tiab] OR "epidemiology report*"[tiab] OR "epidemiology stud*"[tiab] OR "epidemiological report*"[tiab] OR "epidemiological stud*"[tiab] OR "epidemiologic research"[tiab] OR "epidemiological research"[tiab] OR "Epidemiologic Studies"[MAJR] OR "Cohort Studies"[MAJR] OR "Epidemiologic Studies"[Mesh] OR "Cohort Studies"[Mesh]) | 15,725,546 |
| **8** | (#6 AND #7) AND ((2019/11/1:2023/12/31[pdat]) AND (english[Filter])) | 618 |

**Web of Science Core Collection:** Science Citation Index Expanded (SCI-EXPANDED), Social Sciences Citation Index (SSCI), Emerging Sources Citation Index (ESCI)

| **Set #** | **Search** | **# of results** |
| --- | --- | --- |
| **1**  **COVID-19** | TS=(COVID* OR coronavirus* OR "corona virus*" OR "SARS CoV 2" OR SARSCoV2 OR SARSCoV-2 OR "2019 nCoV" OR 2019nCoV OR "nCoV 19" OR "nCoV 2019" OR "severe acute respiratory syndrome coronavirus 2") OR TMIC=("1.104.1353 Coronavirus") | 482,471 |
| **2**  **NPIs** | TS=("non-pharmacological intervention*" OR "nonpharmacological intervention*" OR "nonpharmacologic intervention*" OR "non-pharmacologic intervention*" OR "nonpharmaceutical intervention*" OR "non-pharmaceutical intervention*" OR NPI OR NPIs OR "social distancing*" OR "social separation*" OR "social restrict*" OR "social isolation" OR "physical distancing*" OR "physical separation*" OR "spatial distancing*" OR "spatial separation*" OR "spacial separation*" OR "contact reduction*" OR "contact avoidance" OR ((house* OR home* OR self*) AND (confin* OR quarantin* OR isolat*)) OR "travel restriction*" OR "travel ban" OR "travel bans" OR "banned travel" OR "mobility restriction*" OR "lockdown*" OR "lock down*" OR "shelter in place" OR "shelter in home" OR "stay in home" OR "stay at home" OR "remain in home" OR "remain at home" OR ((school* OR college* OR university* OR business* OR workplace* OR office* OR worksite* OR "work site*") AND (closure* OR closing* OR closed OR cancel* OR suspend* OR suspension*)) OR "remote work*" OR "work at home" OR "work from home" ((gathering* OR crowd* OR "public event*" OR "public activit*" OR "group event*" OR "group activit*" OR "social event*" OR "social activit*" OR "nonessential activit*" OR "non essential activit*" OR "social mixing" OR gastronomy OR restaurant*) AND (restrict* OR limit* OR cancel* OR reduc* OR postpon* OR ban OR bans OR banned OR banning OR suspend* OR suspension*)) OR "border clos*" OR "community containment" OR "contagion mitigat*" OR "containment measure*" OR "suppression measure*" OR "mitigation measure*" OR ((stringen* OR strict* OR enforcement* OR enforcing OR mandatory) AND (mandate* OR policy OR policies OR order* OR law OR laws OR directive*))) | 368,764 |
| **3**  **Transmission Prevention + Precautions** | TS=((((transmission* AND (droplet* OR aerosol* OR airborne OR "air contaminat*")) AND (prevent* OR precaution* OR protect* OR reduc* OR decreas*))) OR "cough etiquette" OR mask* OR "N95 respirator*" OR "N95 mask*" OR "hand wash*" OR "handwash*" OR "hand hygiene" OR "hand disinfect*" OR "hand sanitiz*" OR "hand sanitis*" OR "air filtration" OR "air filter*" OR "HEPA filter*" OR "air purif*" OR "air ventilat*" OR "air recirculat*") | 189,141 |
| **4**  **Contacts** | TS=(transmission* OR transmissib* OR "community spread*" OR superspread* OR super-spread* OR "infectious contact*" OR "contact pattern*" OR "contact mapping" OR "mixing pattern*" OR "incubation period") | 1,150,218 |
| **5**  **Basic Reproduction Number** | TS=(“reproduction number" OR "reproductive number" OR "R_0" OR "R0" OR "R-0" OR "R_0"_ OR "R*_0_*" OR Beta_t OR "effective contact rate*" OR "contact trac*" OR "transmission tracing*" OR "trace strateg*" OR "contact survey*" OR "contact matrices" OR "contact matrix" OR "contact mapping" OR "reduction estimate" OR CoMix OR Polymod OR "susceptible exposed infectious recovered" OR SEIR* OR "susceptible exposed asymptomatic infectious recovered" OR SEAIR* OR "compartmental model*" OR (model* AND (outbreak* OR epidemic* OR pandemic*)) OR "predictive model*" OR forecast*) | 678,443 |
| **6**  **Study Type** | TS=("comparative study" OR "evaluation study" OR "government publication" OR "meta-analysis" OR "observational study" OR review OR "systematic review" OR "technical report" OR "epidemiologic report*"[tiab] OR "epidemiologic stud*"[tiab] OR "epidemiology report*"[tiab] OR "epidemiology stud*"[tiab] OR "epidemiological report*"[tiab] OR "epidemiological stud*"[tiab] OR "epidemiologic research"[tiab] OR "epidemiological research"[tiab]) | 3,552,178 |
| **7** | #1 AND (#2 OR #3) AND #4 AND #5 AND #6 | 336 |
| **8** | #7  Limits: 2019-2023; English; Article, Review, Early Access | **317** |
